# Preparation and Characterization of Narrow Size Distribution PMSQ Microspheres for High-Frequency Electronic Packaging

**DOI:** 10.3390/ma14154233

**Published:** 2021-07-29

**Authors:** Guodong Meng, Yimeng Li, Zhengdong Wang, Cheng Pan, Wenwu Gao, Yonghong Cheng

**Affiliations:** 1 State Key Laboratory of Electrical Insulation and Power Equipment, Xi’an Jiaotong University, Xi’an 710049, China; lym_98415@stu.xjtu.edu.cn (Y.L.); gwenwu@stu.xjtu.edu.cn (W.G.); cyh@xjtu.edu.cn (Y.C.); 2 School of Mechanical and Electrical Engineering, Xi’an University of Architecture and Technology, Xi’an 710055, China; wzd804034945@163.com; 3 School of Electrical Engineering and Automation, Wuhan University, Wuhan 430072, China; pancheng2036@gmail.com

**Keywords:** polymethylsilsesquioxane microspheres, narrow size distribution, sphericity, thermal stability, high-frequency dielectric properties

## Abstract

Polymethylsilsesquioxane (PMSQ) has become a kind of widely studied filler used in the electronic circuit board substrates due to its organic–inorganic hybrid structure, low dielectric constant, and good thermal stability, among other factors. Herein, the PMSQ microspheres were prepared by a two-step acid–base-catalyzed sol–gel method; the influences of reaction conditions including the ratio of water/methyltrimethoxysilane (MTMS), reaction temperature, concentration of the catalyst, and stirring time were systematically investigated; and the optimized reaction condition was then obtained towards a narrow particle size distribution and good sphericity. The microstructure of PMSQ microspheres was analyzed by the infrared spectrum and X-ray diffraction (XRD), which indicated that the as-prepared PMSQ had a ladder-dominant structure. The thermogravimetric analysis (TGA) demonstrated an excellent thermal stability of as-prepared PMSQ microspheres. More specifically, the dielectric constants at high frequency (1~20 GHz) of as-prepared PMSQ microspheres were measured to be about 3.7, which turned out a lower dielectric constant compared to SiO_2_ powder (≈4.0). This study paves the way to further improve the performance of the electronic circuit board substrates for the application of high-frequency electronic packaging.

## 1. Introduction

With the increase of consumer demand in telecommunication devices and computers for cost-effective product miniaturization, the electronic industry urgently needs to find solutions to these problems. These denser and faster microelectronic circuits require that the printed circuit boards (PCBs) operate at high frequency (>1 GHz) with better dielectric properties [1,2]. The conventional insulating substrate in PCBs is glass fiber-reinforced epoxy resins (FR-4) due to their low price, moisture resistance, relative temperature resistance, and good performance at RF/microwave frequencies [3]. However, for high-speed digital circuits or high-frequency analogue applications above a few GHz, they are a poor choice because of their poor dielectric properties (the dielectric constant is greater than 4) at microwave frequencies. For the improvement of some performances such as thermal conductivity, the typical fillers such as silica and alumina are used in FR-4 PCBs, but these dielectric constants of these fillers are not less than 4. Thus, it is necessary to find a new filler with the dielectric constant less than 4 to replace them [3,4,5,6,7,8]. Recently, polysilsesquioxane (PSQ) microsphere is emerging as a new kind of organic–inorganic hybrid particle with the structure formula RSiO_1_._5_, where the R group can be the organofunctional derivatives such as methyl, phenyl, and mercapto, and its architecture can be classified as closed-cage compounds, open-cage structures, ladder structures, and random structures [9]. Due to the organic–inorganic hybrid structure, the PSQ microsphere exhibits both the properties of inorganic silica (i.e., good thermal stability, solvent resistance, and high hardness), and the distinct properties modulated by the diverse surface organofunctional groups such as excellent hydrophobicity and the compatibility with the polymer matrix. Therefore, it has been widely used in the fields of inorganic nanofillers, energy storage, drug carriers, treatment materials for pollution control, UV screening agents, etc. [10,11,12].

Since Stöber, Fink, and Bohn developed the technique of synthesizing monodisperse silica spheres in a water/ethanol solution with silicon alkoxides as the precursor and ammonia as the catalyst [13], a number of methods have been put forward to simplify the reaction system by selecting various precursors and solvents [14,15,16,17]. In essence, the morphology and size of PSQ spheres are quite dependent on reaction conditions, i.e., pH value, reaction time, reaction temperature, stirring speed, and water/monomer ratio. For instance, Tian-Song Deng et al., synthesized vinyl hybrid silica particles through sol–gel reaction and found that the particle size increased as the concentration of organosilane increased while decreasing as the concentration of catalyst increased [16]. Chul Oh et al., reported the highly monodispersed hybrid silica particles with hydroxyl and thiol groups, and discovered a similar relationship between the particle size and the concentration of organosilane and catalyst [18]. Jianbo Yin et al., found that the size of the functionalized silica spheres demonstrated a minimum value with the increase of reaction temperature, while Yong-Geun Lee et al., found that the size of the PSQ spheres decreased with the increase of reaction temperature [14,19]. Moreover, Jiangbo Wang et al., investigated the influence of different reaction conditions such as water/methyltrimethoxysilane (MTMS) ratio, pH value, and condensation reaction temperature, and found that the average size of PSQ microspheres increased with the increase of the reaction temperature, and the larger PSQ microspheres were obtained at smaller pH value [20].

As one of the typical PSQ particles, polymethylsilsesquioxane (PMSQ), which can be prepared through two-step sol–gel synthesis, has been extensively studied due to low dielectric constant, great thermal stability, and high crack resistance, which demonstrates a great potential as filler materials in PCB substrates [21]. Bo Yao et al., found that EVA/PMSQ hybrid, which disperses the obtained PMSQ microsphere in the ethylene-vinyl acetate copolymer (EVA) matrix, showed the improved thermal stability and crystallization ability by using of the strong hydrophobicity of PMSQ [22]. Chaoshuai Lei et al., prepared PMSQ aerogels using a sol–gel system with supercritical ethanol drying, which showed outstanding properties related to the surface area and flexibility, which can be controlled by changing the ammonia concentration [23]. Wenshi Ma et al., prepared the highly monodisperse methyl-functionalized, vinyl-functionalized, and thiol-functionalized polysilsesquioxane spheres (MPSQ, VPSQ, and MPPSQ spheres, respectively) through a one-pot emulsion approach, and found that the organofunctional groups played a key role in thermostability and hydrophobicity of polysilsesquioxane spheres [24]. In terms of permittivity in particular, Jin Kyu Lee et al., prepared PMSQ thin films with dielectric constant of 2.7 at 1 MHz in refluxing THF solutions under nitrogen atmosphere in the presence of HCl catalyst [25]. Takahiro Gunji et al., also prepared the PMSQ via hydrolytic polycondensation with tetramethylammonium hydroxide as the base catalyst, and the dielectric constant of as-prepared coating films by dip coating was evaluated to be 2.6 at 10 kHz [26]. Kai Xi et al., prepared a class of PMSQ films with ultra-low dielectric constant of 1.6 at 1 MHz by using T_8_(Me_4_NO)_8_ polyhedral oligomeric silsesquioxanes (T_8_ POSS) as double-effective porogen [27]. Wen Pin Chuang et al., prepared a series of silane-grafted PMSQ with a controllable structure and molecular weight by grafting tetraethoxysilane (TEOS) and 1H,1H,2H,2H-perfluorodecyltriethoxysilane (PFDTS) on the PMSQ, finding that the molecular structure changed from regular to an irregular random network structure and the RI at 583 nm decreased from 1.50 to 1.42 [28]. Bong Jun Cha et al., realized nanoporous low-k thin (2.12) films with mechanically robust properties using adamantylphenol porogens chemically linked to the PMSQ matrix [29]. He Seung Lee et al., synthesized a series of high molecularweight PMSQs by polycondensation of tetraol, hydroxylsubstituted methylcyclotetrasiloxane, and the dielectric constant of thin film was 2.74 at 1 MHz [30]. Additionally, it is worth noting that although polyhedral oligomeric silsesquioxane (POSS) with a typical cage structure has been considered as an effective way for introducing porous structure to polymers that can obtain a lower dielectric constant, polysilsesquioxane (PSQ) synthesized via sol–gel process has been considered as a special material with low cost and high performance, for example, adjusting the proportion of cage structure and ladder structure of PMSQ, analysis from the infrared spectrum, can obtain the needed mechanical property and dielectric properties [31].

Therefore, most of the previous research work has focused on reducing dielectric constant of PMSQ at low frequency, but ignores the influence mechanism of reaction conditions on the particle size distribution and sphericity, as well as the high frequency dielectric property above 1 GHz, which is very essential to the performance evaluation at high-frequency application. Hence, in this work, polymethylsilsesquioxane (PMSQ) microspheres were prepared by a two-step acid−base-catalyzed sol–gel method, and different reaction conditions including the ratio of water/MTMS, reaction temperatures, concentration of the catalyst, and stirring time were investigated systematically. The chemical structure of as-prepared PMSQ microsphere was characterized by the infrared spectrum and XRD, and, for the first time, the thermal property and high-frequency dielectric property of PMSQ microspheres between 1 and 20 GHz were analyzed as well.

## 2. Materials and Methods

### 2.1. Materials

The precursor methyltrimethoxysilane (MTMS, 99.9%) was purchased from Shanghai Aladdin Bio-Chem Technology Co., LTD. (Shanghai, China); the catalysts acetic acid (≥99.5%) and ammonia (≥99.5%), which were used for preparing 5% acetic acid and 5% ammonia, were purchased from Tianjin Fuyu Fine Chemical Co., LTD. (Tianjin, China), and Tianjin Binhai Cody Chemical Reagent Co., LTD. (Tianjin, China), respectively. All chemicals were used as received without further purification. Deionized water (18.2 M·cm) was used directly from a Milli-Q water system.

### 2.2. Synthesis

**Figure 1:**
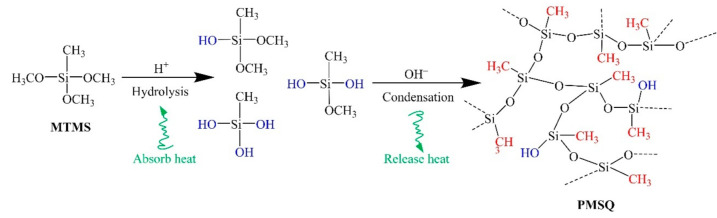
Reaction scheme for the synthesis of PMSQ microspheres.

Figure 1 shows the reaction scheme of the two-step acid−base-catalyzed sol–gel method for PMSQ microsphere synthesis. The first step is an endothermic hydrolysis reaction in which the MTMS is hydrolyzed, wherein some molecules with hydroxyl groups bonded to Si atoms are produced. The second step is an exothermic condensation reaction, in which the final PMSQ structure is formed. Generally, the ultimate morphology is determined by the reaction conditions during hydrolysis and condensation reactions, such as water/MTMS ratio, reaction temperature, stirring time, and pH value, which will be discussed in further detail below.

**Figure 2:**
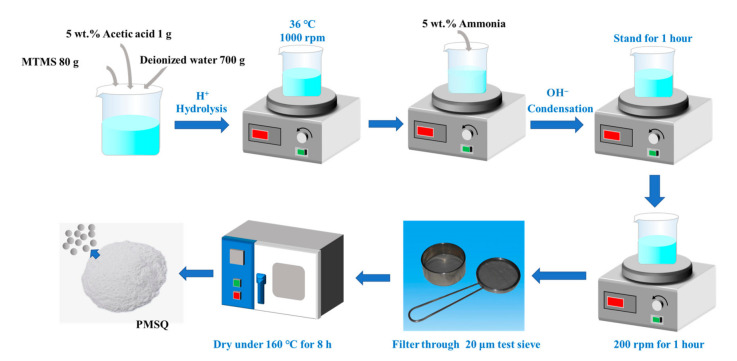
Basic synthesis procedure of the PMSQ microspheres.

Figure 2 illustrates the basic synthesis procedure of PMSQ microsphere by a two-step acid–base-catalyzed sol–gel method. First, MTMS (80 g), deionized water (700 g), and acetic acid (5 wt %, 1 g) were mixed by 1000 rpm stirring at 36 °C until the oil droplets completely disappeared and a transparent solution was obtained. Then, an appropriate amount of ammonia (5 wt %) was added to the solution and stirring was continued until the solution became turbid. After standing for 1 h, the solution was stirred again at 200 rpm for 1 h. Afterward, the solution was filtered by a stainless-steel test sieve with the aperture size of 20 μm, and dried in an oven at 160 °C for 8 h. Finally, the PMSQ microspheres were prepared and fully dispersed to powder. The influences of water/MTMS ratio (500:100, 700:100, 700:80), reaction temperatures (10, 25, 36, 45, 60 °C), concentration of the base-catalyst (which can be equivalent to pH = 11.3, 11.7, 12.0), and stirring time (2, 8, 25 s) on the morphology of PMSQ microspheres were systematically investigated.

### 2.3. Characterization

The morphology of the PMSQ microspheres was observed by scanning electron microscope (SEM; VE-9800S, KEYENCE Co., Osaka, Japan). The particle size and distribution of the PMSQ microspheres were measured by laser particle size analyzer (LS13-320, Beckman Instruments, Inc., Miami, FL, USA). For convenience, we define the particle size uniformity *S*_u_ by D_90_/D_10_, where D_90_ and D_10_ respectively represent 90% and 10% of the volume lying below these values. *S*_avg_ represents the average particle size. The spherical degree *D*_sp_ is defined as the ratio of the equivalent diameter of the particle area to the equivalent diameter of the particle perimeter as follows [32]:

(1) $$D_{\mathrm{sp}}=d_{a}/d_{p},$$

where *d*_p_ represents the equivalent diameter of the particle perimeter *P* (*P* = 2π*d*_p_), and *d*_a_ represents the equivalent diameter of the particle area *A* (*A* = π*d*_a_^2^). Thus, *D*_sp_ = 1 means that the particle is a perfect spherical structure.

The infrared spectrum of the PMSQ microspheres was obtained by FT-IR Spectrometer (VERTEX70, Bruker Co., Ettlingen, Germany). The crystal structure of the PMSQ microspheres was identified by X-ray diffraction (D8 ADVANCE A25, Bruker Co., Karlsruhe, Germany). The thermal degradation behaviors of the PMSQ microspheres were obtained by thermogravimetric analyzer (METTLER TOLEDO TGA/DSC3, Mettler Toledo Co., Nänikon, Switzerland) at a heating rate of 10 °C/min in nitrogen atmosphere. The high-frequency dielectric properties from 1 to 20 GHz were measured by vector network analyzer (Keysight Technologies Inc., Santa Rosa, CA, USA). 

## 3. Results and Discussion

### 3.1. Morphology-Controlled Synthesis of PMSQ Microspheres

The suitable filler particles shall meet the requirements of narrow particle size distribution, good sphericity (0.7~1.0), and average particle size between 1 and 8 μm [32]. For particle fillers used in the PCB substrate, the better sphericity (0.7~1.0) means the better loading property and flow ability, and the narrower particle size distribution and proper particle size (1~8 µm) mean better loading property, anti-flash property, and flow ability [32]. Hence, the essential objective of this work was to synthesize PMSQ microspheres with good sphericity, narrow particle size distribution, and suitable particle size, and obtain the optimized reaction condition by regulating different reaction conditions (i.e., water/MTMS ratio, reaction temperature, stirring time, and pH value).

#### 3.1.1. Influence of Water/MTMS Ratios

**Figure 3:**
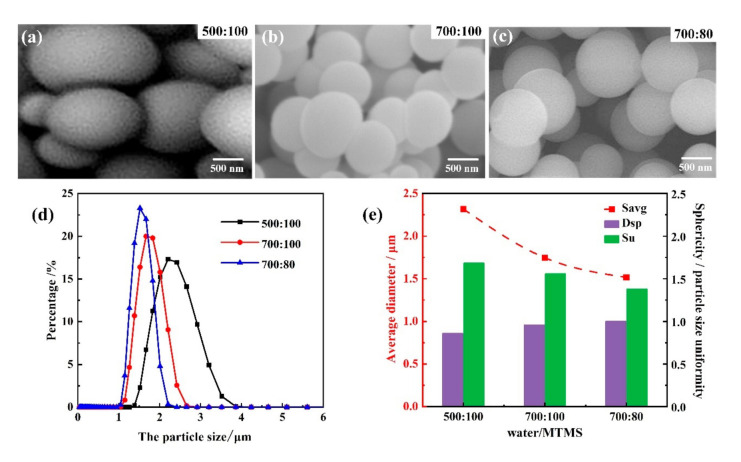
Morphology and particle size distribution of as-prepared PMSQ microspheres with different water/MTMS ratios. (**a**) SEM image of PMSQ microspheres with water/MTMS ratio 500:100. (**b**) SEM image of PMSQ microspheres with water/MTMS ratio 700:100. (**c**) SEM image of PMSQ microspheres with water/MTMS ratio 700:80. (**d**) Particle size distribution of PMSQ microspheres, and (**e**) average diameter, sphericity, and particle size uniformity of PMSQ microspheres with different water/MTMS ratios.

Figure 3 shows the SEM images and morphology parameters of as-prepared PMSQ microspheres with different water/MTMS ratios (500:100, 700:100, and 700:80) under the same experimental conditions of reaction temperature 36 °C, base-catalyst 25 g, and stirring time 8 s. It can be seen that the PMSQ microspheres under 700:100 and 700:80 showed good sphericity degrees that can be quantified by 0.956 and 0.998, respectively, but the sphericity of PMSQ microspheres under 500:100 water/MTMS ratio were poorer by 0.856. As the water/MTMS ratio increased, the average particle size of PMSQ microspheres decreased and they were all in the appropriate range (2.32, 1.75, 1.52 µm), as shown in Figure 3e. Although the PMSQ microspheres under 700:100 water/MTMS showed a good sphericity and average particle size, the particle size distribution and uniformity (1.56) was much worse compared to PMSQ microspheres under 700:80 water/MTMS (1.38), as well as significant agglomeration phenomenon (as shown in Figure 3b). Therefore, the proper water/MTMS ratio was 700:80, where PMSQ microspheres were with the good sphericity, best particle size uniformity, and proper average particle size.

The influence of water/MTMS ratios on the as-prepared PMSQ microspheres can be explained below. As the main reactant of this reaction system, MTMS undergoes hydrolysis and condensation reactions by the catalytic action of acetic acid. During the hydrolysis reaction, attack of the water protons will promote the nucleophilic addition of the water molecule. As a result, the more water there is, the greater the possibility of H^+^ nucleophilic attack on Si, and the faster rate of the hydrolysis reaction as well [22]. Under the faster rate of the hydrolysis reaction, more crystal nuclei are generated at the critical nucleation concentration, and the smaller average particle size of PMSQ microspheres will be. During the condensation reaction, Si–O–Si bonds and water are generated. The water acts as both solvent and polymerization inhibitor. On one hand, the particle size becomes smaller as the increase of water, since the reaction mass is diluted. Thus, the condensation reaction rate is reduced, and the polymerization of silanol monomer proceeds in a smaller range. On the other hand, the hydrogen in the water molecule seizes the oxygen atom on the hydroxyl group in Si–OH to form a hydrogen bond to hinder the reaction of Si–OH, thereby inhibiting the polymerization to a certain extent, which also prevents the particle size from increasing. Therefore, the average particle size of PMSQ microspheres decreases with the water/MTMS ratio increases.

#### 3.1.2. Influence of Reaction Temperatures

**Figure 4:**
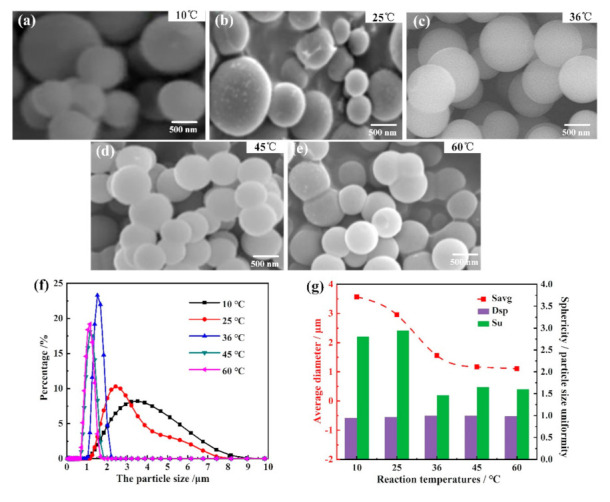
Morphology and particle size distribution of as-prepared PMSQ microspheres with different reaction temperatures. (**a**–**e**) SEM images at different reaction temperatures; (**f**) the particle size and distribution of the PMSQ microspheres; and (**g**) average diameter, sphericity, and particle size uniformity of PMSQ microspheres with different reaction temperatures.

Figure 4 shows the SEM images and morphology parameters of as-prepared PMSQ microspheres of as-prepared PMSQ microspheres with different reaction temperatures (10, 25, 36, 45, 60 °C) under experimental conditions of 700:80 water/MTMS, 25 g base-catalyst, and 8 s high-speed stirring time. It can be seen that the degrees of sphericity of PMSQ microspheres under five reaction temperatures were 0.949, 0.968, 0.998, 0.994, and 0.982, respectively. As the reaction temperature increased, the average particle size decreased sharply from 3.57 µm under 10 °C to 1.52 µm under 36 °C, and then decreased gently from 1.52 to 1.11 µm under 60 °C, all meeting the requirements of particle size. Nevertheless, the prepared particles demonstrated a broad size distribution and worse particle size uniformity (2.81 and 2.94) when reaction temperatures were 10 and 25 °C, and although the prepared particles showed a relatively narrow size distribution (1.65 and 1.60) under 45 and 60 °C, the obvious agglomeration can be observed from SEM images. Thus, the appropriate reaction temperature was 36 °C where the particle size uniformity (1.38) and sphericity were the best. 

The influence of reaction temperatures on the as-prepared PMSQ microspheres can be explained below. The formation of PMSQ microspheres is the result of the combined action of MTMS hydrolysis reaction and condensation reaction, in which hydrolysis absorbs heat and condensation releases heat. Under the premise that the hydrolysis and the condensation reactions reach a dynamic equilibrium, the particle size will become smaller at either a larger hydrolysis rate or a smaller condensation rate. Therefore, the increase of the reaction temperature will provide more heat and accelerate the hydrolysis reaction, which could produce a large number of crystal nuclei according to the Arrhenius equation as follows:

*k* = *A* exp *(−E/RT)* (2)

where *k* is the rate of chemical reaction, *A* is a constant depending on the chemicals involved, *E* is the activation energy, *R* is the universal gas constant, and *T* is the absolute temperature [33]. At the same time, the increases of collision probability between particles will also increase the condensation reaction rate. It is worth noting that the hydrolysis rate was greater than the condensation rate, which led to a sharp increase in the number of crystal nuclei and thus a downward trend regarding the microsphere size. On the other hand, since the condensation reaction is an exothermic reaction, the increase of reaction temperature will hinder the condensation reaction and inhibit the particle growth according to the Le Chatelier’s principle [34]. However, higher temperature (>36 °C) will result in too many crystal nuclei, and therefore there would be insufficient space for the free growth of particles, and the agglomeration cannot be avoided. Therefore, the optimal reaction temperature was found to be 36 °C.

#### 3.1.3. Influence of the Base Catalyst Concentrations

**Figure 5:**
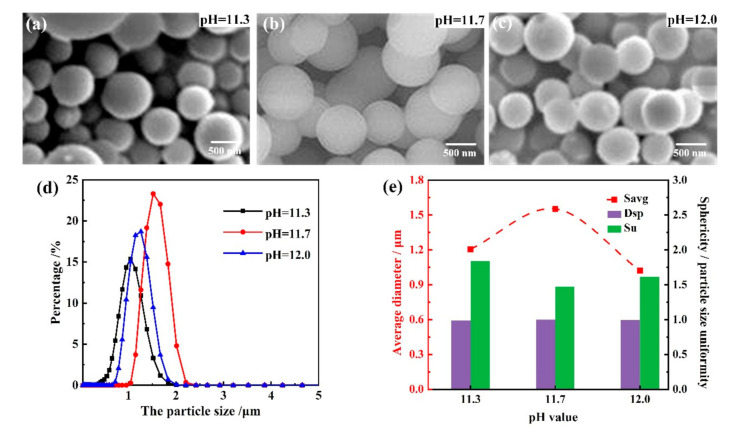
Morphology and particle size distribution of as-prepared PMSQ microspheres with different base catalyst concentrations. (**a**–**c**) SEM images; (**d**) the particle size and distribution of the PMSQ microparticles; and (**e**) average diameter, sphericity, and particle size uniformity of PMSQ microspheres with different base catalyst concentrations.

Figure 5 shows the SEM images and morphology parameters of as-prepared PMSQ microspheres with different base catalyst concentrations (which can be represented by the pH values 11.3, 11.7, and 12.0), under experimental conditions of 700:80 water/MTMS, 36 °C reaction temperature, and 8 s high-speed stirring time. It can be seen that the sphericity degrees under pH = 11.3, 11.7, and 12.0 were 0.986, 0.998, and 0.991, respectively, which means a good conglomeration rate regardless of base catalyst concentrations. As the pH value increased, the average particle size changed a little from 1.21 to 1.52 µm, and then from 1.52 to 1.02 µm. The obvious differences between these three groups were the particle size distribution and uniformity, wherein the PMSQ microspheres under pH = 11.7 showed the best uniformity of 1.38 compared to the 1.84 at pH = 11.3 and 1.62 at pH = 12.0. Hence, the satisfied pH value was 11.7 with the best particle size uniformity.

The influence of different base catalyst concentrations on the as-prepared PMSQ microspheres can be explained below. During the condensation process, OH^−^ of the alkaline catalyst (NH_4_OH) will directly attack silicon atom and cause the silicon atomic nuclei to be charged negatively due to its small radius, and then H^−^ will break away from the Si–OH bond, which could promote the condensation process. Since the electron cloud density around the silicon atom and the steric hindrance effect of H^−^ have a greater influence on the condensation reaction, the replacement of H^−^ becomes easier as the -OH around the silicon atom decreases. Thus, in the condensation reaction of silanol, the Si–OH condensation rate is slow in the beginning, but the subsequent condensation rate is accelerated and finally PMSQ microspheres are formed. As the OH^−^ concentration in the solution increases, the condensation rate of the monomer and the particle size both increases, but the hydrolysis rate and the number of generated cores increase additionally. Therefore, the final size of the particles decreases when the content of MTMS is constant. The research on the degree of dispersion show that lower ammonia concentration will lead to a large degree of dispersion, which is not good for the growth of particles, while higher ammonia concentration can also result in too many crystal nuclei, and there is no sufficient space for the free growth of particles and the agglomeration cannot be avoided.

#### 3.1.4. Influence of High-Speed Stirring Time

**Figure 6:**
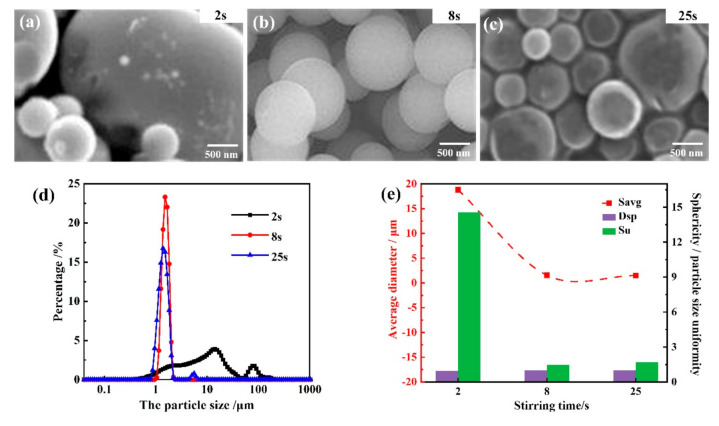
Morphology and particle size distribution of as-prepared PMSQ microspheres with different high-speed stirring times. (**a**–**c**) SEM images; (**d**) the particle size and distribution of the PMSQ microparticles; and (**e**) average diameter, sphericity, and particle size uniformity of PMSQ microspheres with different high-speed stirring times.

Figure 6 shows the SEM images and morphology parameters of as-prepared PMSQ microspheres with different high-speed stirring times from adding ammonia (2 s, 8 s, 25 s), under experimental conditions of 700:80 water/MTMS, 36 °C reaction temperature, and 25 g base catalyst. It can be seen that the sphericity degrees of PMSQ microspheres under three high-speed stirring times (2 s, 8 s, 25 s) were 0.941, 0.998, and 0.998, respectively. The shorter stirring time (i.e., 2 s) caused a much larger average particle size of 18.8 μm in a wide range from 0.048 to 234 μm, and the average particle size was approximately 1.5 μm when the stirring time was set to 8 s or 25 s. It can also be seen from the particle size uniformity that the particle size uniformity was 14.6 under 2 s stirring time compared to 1.68 under 25 s and 1.38 under 8 s. Thus, the proper high-speed stirring time was 8 s, wherein the PMSQ microspheres showed the best sphericity and particle size uniformity.

The influence of different high-speed stirring times on the as-prepared PMSQ microspheres can be explained below. Since the magnetic beads exert a certain impact, shear, and other forces on the silanol during the solution stirring process, the longer the stirring time is, the longer the intermediate silanol bears. The time from adding ammonia to making the solution cloudy during the preparation process is about 8 s. The stirring time is too short to allow the ammonia to fully contact the solution to exert its catalytic effect, and the uneven mixing of ammonia and solution will result in poor particle size uniformity of the generated PMSQ microspheres. In addition, the particle growth process after nucleation requires a stable force field environment, and therefore excessive stirring time will destroy this environment, which will not only increase the chance of silanol monomers colliding under condensation and producing particles with large particle size, but will also cause the change of force field, which will cause the particle sphericity to become worse.

#### 3.1.5. Summary of the Influence of Different Reaction Conditions on the PMSQ Microspheres

Table 1 summarizes the morphology parameters of as-prepared PMSQ microspheres under different reaction conditions. As we can see, the optimized reaction condition turned out to be the ratio of water/MTMS 700:80, reaction temperature 36 °C, pH = 11.7, and stirring time 8 s, wherein the PMSQ microspheres showed good sphericity of 0.998, particle size uniformity of 1.38, and proper average particle size of 1.52 µm, which satisfy the requirements of particle fillers in PCB. In addition, the yield of PMSQ microspheres prepared at this optimal condition was about 85% according to the total weight of microspheres and raw material.

### 3.2. Structure of the PMSQ Microparticles

**Figure 7:**
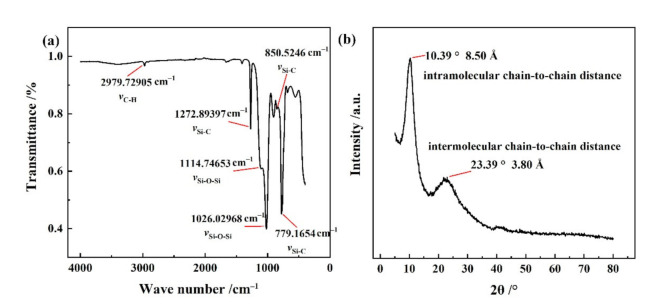
The chemical structure characterization of as-prepared PMSQ microparticles. (**a**) FT-IR spectrum, (**b**) XRD pattern.

Figure 7 shows the chemical structure characterization of as-prepared PMSQ microparticles under the optimized experimental condition. Figure 7a shows the FT-IR spectrum of as-prepared PMSQ microspheres. According to previous literature, the absorption band at 2979 cm^−1^ corresponds to asymmetrical stretching vibration of methyl group (-CH_3_) [28,35]. The absorption band at 1272 cm^−1^ is characteristic of the stretching vibration of Si–CH_3_, and the bands of absorption at 779 and 850 cm^−1^ correspond to the out-of-plane agitation of methyl group in Si–CH_3_. The absorption bands at 1026 and 1114 cm^−1^ are the asymmetrical Si–O–Si stretching mode, which can be extended into a higher frequency band and a lower frequency band, depending of the stretch vibration mode in the (Si–O)_n_ ring subunit. More importantly, these two peaks are good indicators for a cage/ladder structure [35]. The ladder polysilsesquioxane was synthesised firstly in 1960 by Brown group, and the IR spectroscopy could monitor the interconversion between cube and ladder polyphenylsilsesquioxane (PPSQ), wherein the cube structure showed one strong absorption band at 1120–1130 cm^−1^, while two absorptions were found at 1150–1135 cm^−1^ and 1045 cm^−1^ for ladder polymers, regardless of the side group [36]. Thus, it can be seen from Figure 7a that the absorption band at 1026 cm^−1^ was sharper than that at 1114 cm^−1^, indicating the ladder structure was dominant [37,38,39,40].

Figure 7b shows the XRD pattern of the PMSQ microspheres, which exhibited ladder structure due to the presence of two peaks in XRD pattern. The first characteristic halo appeared at 2θ = 10.39°, corresponding to the intramolecular chain-to-chain distance of 8.50 Å according to Bragg’s law. The second characteristic halo appeared at 2θ = 23.39°, indicating that the average intermolecular chain-to-chain distance in the PMSQs was approximately 3.80 Å [20,40]. Therefore, FT-IR spectrum and XRD analysis of the PMSQ microspheres demonstrated that the as-prepared PMSQ had a highly asymmetric structure that well matched the ladder-dominant structure.

### 3.3. Thermal Stability of PMSQ Microspheres

**Figure 8:**
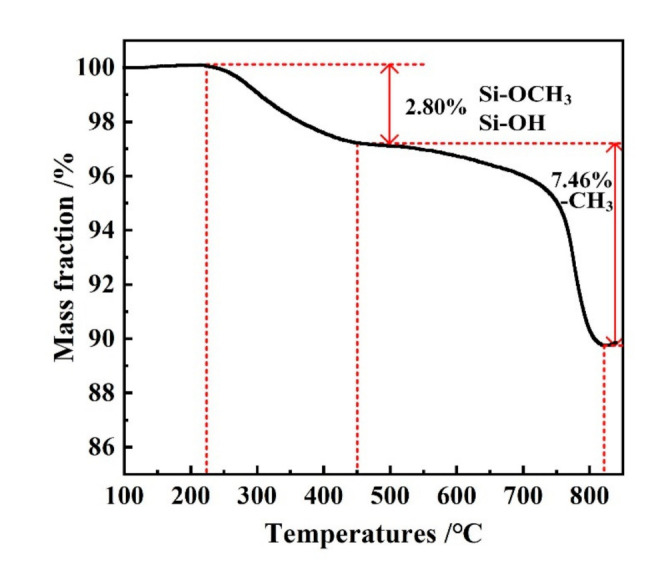
TGA curves of PMSQ microspheres in nitrogen atmosphere.

Figure 8 shows the thermogravimetric analysis (TGA) curve of as-prepared PMSQ microspheres in nitrogen atmosphere. There are two mass-change steps. The first step was a 2.80% weight loss when the temperature increased from 225 to 450 °C, corresponding to the decomposition of residual silanol groups and methoxy groups [24]. The second step was a 7.46% weight loss when the temperature increased from 450 to 825 °C, corresponding to the decomposition of methyl groups on the surface of PMSQ, and therefore the total weight loss of PMSQ from 100 to 825 °C was around 10.26%. As we can see, when the temperature came to 450 °C, there was a little loss (less than 5%) for the PMSQ microspheres, and thus the PMSQ had good thermal stability in the nitrogen atmosphere.

### 3.4. Dielectric Properties of PMSQ Microspheres at High Frequency

**Figure 9:**
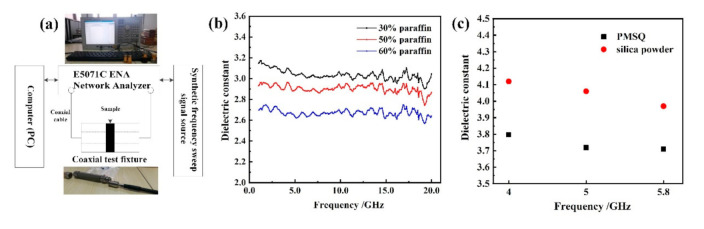
(**a**) Schematic diagram of coaxial transmission line test, (**b**) dielectric constant of PMSQ/paraffin composites with different mass fraction of paraffin, and (**c**) comparison of dielectric constant between PMSQ and silica powder.

In order to measure the dielectric properties of as-prepared PMSQ microspheres at high frequency (1~20 GHz), we adopted the coaxial transmission line method in the experiments. Figure 9a shows the schematic diagram of the coaxial transmission line test system. The principle of measurement is as follows. When the incident electromagnetic wave *V_i_* in the cavity of the transmission line encounters the sample, one part of the electromagnetic wave *V_t_* will transmit through the sample, and the other part *V_r_* will reflect on the sample, during which energy attenuation and phase shift occurs. The electromagnetic characteristics of the sample to be measured are calculated by the relationship between the reflection coefficient, the transmission coefficient, and the scattering parameters, which are measured by the vector network analyzer.

During the test, paraffin wax was used as a standard base material to mix with the sample. Paraffin is a translucent substance, and its melting point is about 70 °C. The paraffin, regardless of whether it is in solid or liquid form, can be very chemically stable and difficult to react with other substances in general. Meanwhile, certain oil content can reduce the hardness of paraffin wax, and therefore paraffin wax can be easily processed into various shapes. Most importantly, paraffin has an extremely stable dielectric spectrum, and the dielectric constant and dielectric loss factor can keep around 2.1 and 0, respectively, even from 1 to 20 GHz. Thus, paraffin is mixed with the as-prepared PMSQ microsphere powder during the transmission reflection test. The preparation procedure of the paraffin/PMSQ composite is as follows. First, the PMSQ to be tested shall be dried appropriately. Second, the accuracy of weighing is ensured. Third, the paraffin wax is heated until it melts, and it is mixed with the powder evenly, and finally the paraffin/PMSQ composites are prepared in varying proportions (30, 50, and 60 wt %) for testing.

Since the imaginary part in the complex permittivity of the PMSQ powders is very small, approximately 0, the analysis of the measurement results only paid attention to the real part in the complex permittivity. Figure 9b shows the dielectric constants of PMSQ/paraffin composites with different paraffin mass fractions as a function of frequency. As the paraffin mass fraction increased, the dielectric constant of PMSQ/paraffin composite decreased, and there was almost no frequency dependency in the permittivity of the PMSQ in the measurement frequency band. Generally, the lower mass fraction of paraffin, the closer the permittivity of composite is to PMSQ microspheres. Here, we adopted the Bruggeman formula to calculate the complex permittivity of the prepared PMSQ [41]:

(3) $$f(\varepsilon_{i}-\varepsilon_{\mathrm{eff}})/(\varepsilon_{i}+2\varepsilon_{\mathrm{eff}})+(1-f)(\varepsilon_{m}-\varepsilon_{\mathrm{eff}})/(\varepsilon_{m}+2\varepsilon_{\mathrm{eff}})=0$$

where *f* is the volume content of PMSQ in the paraffin/PMSQ composite, which can be calculated by the PMSQ density 1.30 g/cm^3^ and paraffin density 0.90 g/cm^3^; *ε*_i_ is the complex permittivity of PMSQ; *ε*_m_ is the complex permittivity of paraffin; and *ε*_eff_ is the complex permittivity of the composite. Figure 9c shows the dielectric constant comparison between the our PMSQ microspheres and silica powders in [42] at 4, 5, and 5.8 GHz. As we can see, the dielectric constants of the prepared PMSQ powder were around 3.7, which is superior to the dielectric constants about 4 of silica powder. Because of the lack of frequency dependency, the dielectric constants of PMSQ powder can be thought to be better than silica powder in the frequency between 1 and 20 GHz. Hence, the PMSQ microspheres are expected to be used in PCB substrate fillers.

## 4. Conclusions

The influences of various reaction conditions on the morphology and particle size distribution of PMSQ microspheres have been investigated, including the ratio of water/MTMS, reaction temperatures, concentration of the catalyst, and stirring time. The FT-IR spectrum and XRD analysis confirmed that the as-prepared PMSQ structure is a ladder-dominant structure, and the optimum conditions for PMSQ microspheres, with good sphericity, narrow size distribution, and good dispersity, can be summarized as ratio of water/MTMS 700:80, reaction temperature 36 °C, pH = 11.7, and stirring time 8 s. The as-prepared PMSQ microspheres also demonstrated an excellent thermal stability. Furthermore, the dielectric constants of optimized PMSQ microspheres were measured to be about 3.7 within the range from 1 to 20 GHz, which is superior to SiO_2_ powder (≈4.0). Therefore, the PMSQ microspheres were successfully obtained, with low dielectric constant, narrow particle size distribution, and good sphericity, which should be a very potential filler candidate for high-frequency electronic packaging at high frequency above 1 GHz.

## Figures and Tables

**Table 1:** Summary of as-prepared PMSQ microspheres via different reaction conditions.

Sample Serial No.	Reaction Condition				Morphology Parameters		
	Water/MTMS (g)	Temperatures (℃)	Acetic Acid/Ammonia (g)	Stirring Time (s)	Sphericity	Average Particle Size (µm)	Particle Size Uniformity
1	700:80	36	1:25	8	0.998	1.52	1.38
2	500:100	36	1:25	8	0.856	2.32	1.68
3	700:100	36	1:25	8	0.956	1.75	1.56
4	700:80	10	1:25	8	0.949	3.57	2.81
5	700:80	25	1:25	8	0.968	2.96	2.94
6	700:80	45	1:25	8	0.994	1.17	1.65
7	700:80	60	1:25	8	0.982	1.11	1.60
8	700:80	36	1:5	8	0.986	1.21	1.84
9	700:80	36	1:100	8	0.991	1.02	1.62
10	700:80	36	1:25	2	0.941	18.8	14.6
11	700:80	36	1:25	25	0.998	1.49	1.68

## Data Availability

The data presented in this study are available in the article.
